# Minimally invasive *versus* open lateral pancreaticojejunostomy in patients with painful chronic pancreatitis: systematic review

**DOI:** 10.1093/bjsopen/zrae135

**Published:** 2025-01-21

**Authors:** Roberto M Montorsi, Michiel F G Francken, Marja A Boermeester, Olivier R Busch, Freek Daams, Thilo Hackert, Roel Haen, Markus W Hollmann, Hjalmar C van Santvoort, Marc G Besselink

**Affiliations:** Department of Surgery, Amsterdam UMC, location University of Amsterdam, Amsterdam, The Netherlands; Department of Surgery, Amsterdam Gastroenterology Endocrinology Metabolism, Amsterdam, The Netherlands; Department of General and Pancreatic Surgery, The Pancreas Institute, University of Verona Hospital Trust, Verona, Italy; Department of Surgery, Amsterdam UMC, location University of Amsterdam, Amsterdam, The Netherlands; Department of Surgery, Amsterdam Gastroenterology Endocrinology Metabolism, Amsterdam, The Netherlands; Department of Research and Development, St Antonius Hospital, Nieuwegein, The Netherlands; Department of Surgery, Amsterdam UMC, location University of Amsterdam, Amsterdam, The Netherlands; Department of Surgery, Amsterdam Gastroenterology Endocrinology Metabolism, Amsterdam, The Netherlands; Department of Surgery, Amsterdam UMC, location University of Amsterdam, Amsterdam, The Netherlands; Department of Surgery, Amsterdam Gastroenterology Endocrinology Metabolism, Amsterdam, The Netherlands; Department of Surgery, Amsterdam Gastroenterology Endocrinology Metabolism, Amsterdam, The Netherlands; Department of Surgery, Amsterdam UMC, location Vrije Universiteit, Amsterdam, The Netherlands; Department of General, Visceral and Thoracic Surgery, University Medical Center Hamburg-Eppendorf, Hamburg, Germany; Department of Surgery, Erasmus University Medical Center, Rotterdam, The Netherlands; Department of Anesthesiology, Amsterdam UMC, University of Amsterdam, Amsterdam, The Netherlands; Department of Surgery, St. Antonius Hospital, Nieuwegein, The Netherlands; Department of Surgery, University Medical Center Utrecht, Utrecht, The Netherlands; Department of Surgery, Amsterdam UMC, location University of Amsterdam, Amsterdam, The Netherlands; Department of Surgery, Amsterdam Gastroenterology Endocrinology Metabolism, Amsterdam, The Netherlands

## Abstract

**Background:**

Patients with painful chronic pancreatitis combined with a dilated main pancreatic duct and a normal size pancreatic head are treated according to guidelines by lateral pancreaticojejunostomy (LPJ). This systematic review compared outcomes of minimally invasive LPJ and open LPJ.

**Methods:**

From 1 January 2000 until 13 November 2023, series reporting on minimally invasive LPJ and open LPJ in patients with symptomatic chronic pancreatitis were included. This study was structured in accordance with the PRISMA guidelines. The primary outcome was intraoperative and postoperative complications. Secondary outcomes included long-term clinical outcomes.

**Results:**

Overall, 19 retrospective studies were included. Morbidity rate ranged from 0% to 57% after minimally invasive LPJ *versus* 4% to 68% after open LPJ (median: 25, i.q.r.: 23). Length of hospital stay ranged from 5 to 7 days after minimally invasive LPJ and from 6 to 16 days after open LPJ. The rate of pain relief ranged from 62% to 91% after open LPJ (median: 78.5, i.q.r.: 23) and from 71% to 100% (median: 82.5, i.q.r.: 12.5) after minimally invasive LPJ respectively. New-onset endocrine insufficiency ranged from 21% to 22% in minimally invasive LPJ and 19% to 26% after open LPJ. New-onset exocrine insufficiency was shown in 11% to 27% in minimally invasive LPJ *versus* 8% to 26% after open LPJ. Weight gain ranged from 60% to 100% (median: 97, i.q.r.: 23) after minimally invasive LPJ.

**Discussion:**

This systematic review suggested that minimally invasive LPJ can be performed safely in selected patients with symptomatic chronic pancreatitis. Phase 2 randomized trials should assess potential short-term benefits such as postoperative pain and length of hospital stay after minimally invasive LPJ.

## Teaser

This systematic review reports on outcomes of minimally invasive (MIS-LPJ) and open lateral pancreaticojejunostomy (LPJ) in 1286 patients with symptomatic chronic pancreatitis.

## Introduction

Chronic pancreatitis (CP) can be characterized by pain, endocrine and exocrine pancreatic insufficiency, and increased risk of pancreatic cancer, resulting in reduced quality of life^1,2^. Management of CP aims to increase patients’ quality of life (QoL) by relieving symptoms and preventing disease progression. Chronic pain in CP is a multifactorial problem^3,4^. Approximately 75% of patients with CP experience chronic pain and 30% of these patients need medical therapy and eventually require intervention^5^.

Several randomized studies have demonstrated that surgery is the most effective treatment, especially in patients with CP and a dilated main pancreatic duct^6–8^. According to current guidelines^9–12^, lateral pancreaticojejunostomy (LPJ) is the procedure of choice in patients with chronic pain and is indicated in patients in whom the main pancreatic duct is dilated (>5 mm) but without an enlargement of the pancreatic head (≤40 mm). Surgical LPJ is associated with a low risk of mortality and a relatively low risk of postoperative complications^9^.

Following surgery, these patients are prone to a challenging pain therapy. Most patients are long-standing opioid users and may present with opioid misuse, abuse, tolerance, dependency, or even opioid-induced hyperalgesia, lowering sensitivity to opioids or, in the case of hyperalgesia, they might even suffer from increased pain following opioid administration. Therefore, minimally invasive surgery (MIS) LPJ has been suggested as a means of minimizing direct postoperative pain, thus enhancing time to functional recovery while reducing length of hospital stay, as long as the effectivity on CP-related pain is as good as open surgery. Large series and systematic reviews comparing MIS and open LPJ are currently lacking.

The aim of this systematic review was to report the surgical and long-term outcomes of MIS compared to open LPJ.

## Methods

This systematic review was structured in accordance with the PRISMA^13^ guidelines. The study protocol was registered with the International Prospective Register of Systematic Reviews^14^ (PROSPERO) on 25 December 2023 (registration number CRD42023493554).

### Information sources and search strategy

A systematic literature search was performed in PubMed, EMBASE (Ovid), and the Cochrane Library. The search included the words (“Pancreatitis”[Mesh] OR “Pancreatic Diseases”[Mesh] OR pancrea*[tiab]) AND (“Minimally Invasive Surgical Procedures”[Mesh] OR “Robotic Surgical Procedures”[Mesh] OR “Robotics”[Mesh] OR “Laparoscopy”[Mesh] OR “Pancreatitis/surgery”[Mesh:NoExp] OR “Pancreatitis, Chronic/surgery”[Mesh] OR robot*[tiab] OR RDP[tiab] OR minimally invasiv*[tiab] OR minimal invasiv*[tiab] OR mini-invasiv*[tiab] OR laparoscop*[tiab] OR open[tiab]) AND (“Pancreaticojunostomy”[MeSH] OR pancreatojejunostom*[tiab] OR pancreaticojejunostom*[tiab] OR pancreato jejun*[tiab] OR pancreatico jejun*[tiab] OR puestow[tiab]) NOT ((“Animals”[MeSH Terms] OR “models, animal”[MeSH Terms] OR “Animal Experimentation”[MeSH Terms] OR rat[tiab] OR rats[tiab] OR mice[tiab] OR mouse[tiab] OR murine[tiab] OR murines[tiab] OR rodent[tiab] OR rodents[tiab] OR rabbit[tiab] OR rabbits[tiab] OR cat[tiab] OR cats[tiab] OR dog[tiab] OR dogs[tiab] OR pig[tiab] OR pigs[tiab] OR cow[tiab] OR cows[tiab] OR monkey[tiab] OR monkeys[tiab] OR goat[tiab] OR goats[tiab] OR horse[tiab] OR horses[tiab] OR ape[tiab] OR apes[tiab] OR gorilla[tiab] OR gorillas[tiab] OR sheep[tiab] OR sheeps[tiab] OR ovine[tiab] OR lamb[tiab] OR swine[tiab] OR swines[tiab] OR porcine[tiab] OR pup[tiab] OR pups[tiab] OR canine[tiab] OR beagle[tiab]) NOT “Humans”[MeSH Terms]) NOT (“Letter” [Publication Type] OR “Comment” [Publication Type] OR “Editorial” [Publication Type] OR letter[ti] OR editorial[ti] OR comment*[ti]) AND (“2000/01/01”[Date—Publication] : “2023/11/13”[Date—Publication]). Duplicates were removed.

### Eligibility criteria

All published studies reporting on MIS and/or open LPJ in patients with chronic pancreatitis were screened for eligibility. Both comparative and non-comparative studies were included. Randomized clinical trials (RCTs), prospective and retrospective observational studies were included (1 January 2000–13 November 2023). Case series reporting on less than five MIS LPJ, trial protocols, systematic reviews, meta-analyses, conference abstracts, secondary publications of previously published studies, letters and commentaries were excluded. Publications lacking full text and publications in languages other than English were also excluded. In order to prevent a much larger control group than intervention group, studies with <50 open LPJ were excluded.

### Data collection process

Two authors (R.M.M. and M.F.G.F.) independently screened literature on title and abstract in a blinded fashion and ultimately on full text using the aforementioned eligibility criteria using Rayyan^15^ (version 2022). Disagreements were resolved by consensus and, if necessary, by the opinion of a third reviewer (M.G.B.).

### Data items

Data extraction included the publication details (for example study title, publication date, authors, study design), baseline characteristics (for example number of patients, sex, age, clinical characteristics, interventions (for example type of surgical approach, intraoperative variables) and postoperative characteristics (for example postoperative pancreatic fistula^16^, delayed gastric emptying^17^, postpancreatectomy haemorrhage^18^, bile leak^19^, chyle leak^20^, postpancreatectomy acute pancreatitis^21^, general postoperative complications, Clavien–Dindo classification^22^, quality of life, long-term outcomes). Primary and secondary endpoints were extracted additionally.

### Outcome

The primary outcomes considered intraoperative (for example operative time, estimated blood loss) and postoperative (for example morbidity rate, any postoperative complication regardless of Clavien–Dindo grade, length of hospital stay, readmission, mortality) data. Secondary outcomes included long-term outcomes (for example pain relief, endocrine insufficiency, exocrine insufficiency, weight gain, medical costs).

### Risk of bias assessment

The quality of the included studies was assessed by two independent reviewers (R.M.M. and M.F.). The methodological index for non-randomized studies (MINORS)^23^ and Risk of Bias 2 (RoB2)^24^ criteria were used for non-randomized and randomized studies respectively. As stated by MINORS, the quality of comparative and non-comparative non-randomized studies was assessed with eight criteria. In case of comparative studies, the additional criteria assessed were the adequacy of the control group, contemporary groups, baseline equivalence of groups, and adequate statistical analyses. Disagreements were resolved by consensus and, if necessary, by the opinion of a third reviewer (M.G.B.).

### Statistical analysis

Descriptive statistics were used to summarize the extracted data. Continuous data were presented as mean with standard deviation or as median with (interquartile) range accordingly. Binary or categorical data were presented as frequencies (%). Based on the quality of the data a formal meta-analysis was not performed but rather medians with interquartile range are presented for percentages whenever possible.

## Results

**Fig. 1:**
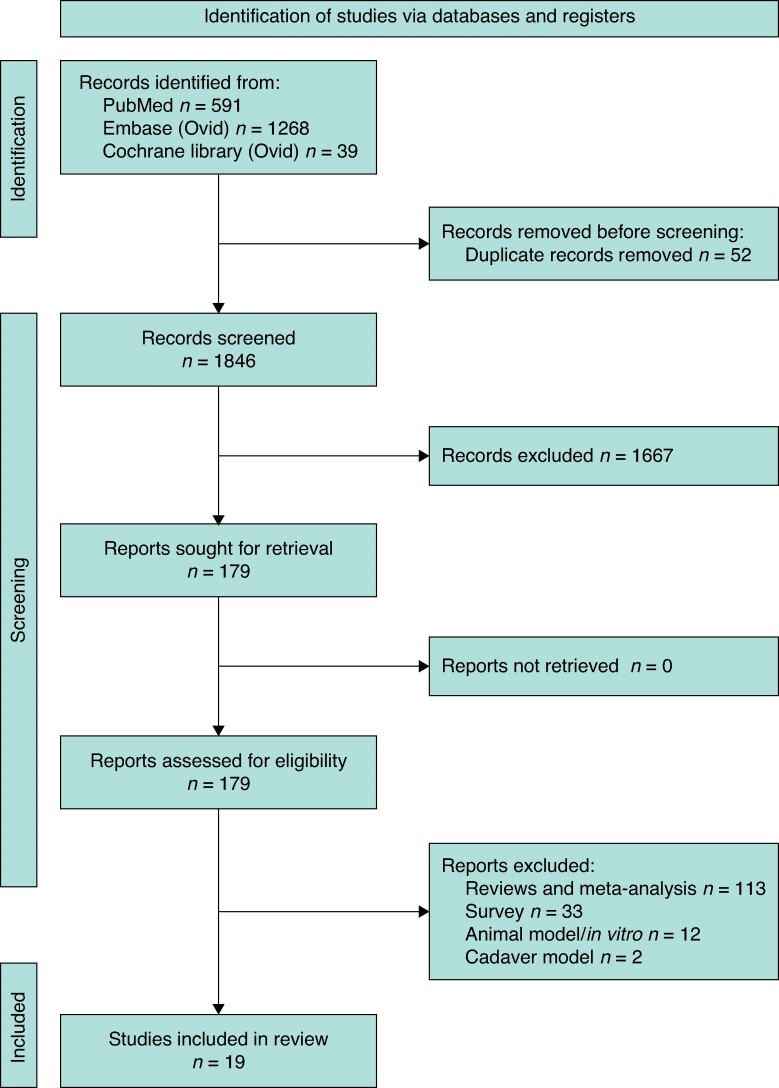
PRISMA 2020 flow diagram for included studies

After screening 1898 studies, 19 studies with 1338 patients fulfilled the eligibility criteria (*Fig. 1*), with 177 (13%) patients after MIS LPJ and 1161 (87%) after open LPJ^25–43^ (*Table 1*). The included studies originated from seven countries (USA, UK, India, the Netherlands, South Korea, France, and Japan). All studies were retrospective in nature, as no prospective observational studies and RCTs were identified. Two studies were comparative studies, whereas 17 were non-comparative. The smallest study was published by Khaled and Ammori^29^ (5 laparoscopic LPJ) and the largest was reported by Napolitano *et al.*^39^ (524 open Puestow LPJ). Outcome of MIS LPJ was reported in eight studies, outcome of open LPJ in nine studies, and two studies^31,32^ compared both approaches. Extracted data are displayed in *Tables 1–3* and *Tables S1–2*.

**Table 1:** Study characteristics on minimally invasive and open lateral pancreaticojejunostomy for symptomatic chronic pancreatitis

Years	Authors	Country	Study design	MIS *versus* open	LPJ	Type of approach	MINORS score
**Minimally invasive LPJ**				(*n* = 132)			
2004	Tantia *et al*.^37^	India	Retrospective	No	17	Laparoscopic	5/16
2006	Palanivelu *et al*.^34^	India	Retrospective	No	12	Laparoscopic	4/16
2014	Khaled and Ammori^29^	UK	Retrospective	No	5	Laparoscopic	8/16
2013	Sahoo and Kumar^35^	India	Retrospective	No	12	Laparoscopic	5/16
2016	Kim and Hong^30^	Korea	Retrospective	No	11	Laparoscopic	5/16
2018	Hamad *et al*.^27^	USA	Retrospective	No	8	Robotic	10/16
2019	Bhandarwar *et al*.^25^	India	Retrospective	No	28	Laparoscopic	9/16
2019	Senthilnathan *et al*.^36^	India	Retrospective	No	39	Laparoscopic	9/16
**Open LPJ**				(*n* = 1104)			
2000	Sielezneff *et al*.^41^	France	Retrospective	No	57	Open	10/16
2000	Sohn *et al*.^42^	USA	Retrospective	No	52	Open	9/24
2001	Nealon and Matin^40^	USA	Retrospective	No	124	Open	8/24
2001	Kalady *et al*.^28^	USA	Retrospective	No	60	Open	7/16
2002	Boerma *et al*.^26^	The Netherlands	Retrospective	No	50	Open	8/24
2003	Nealon and Walser^33^	USA	Retrospective	No	103	Open	8/24
2014	Sudo *et al*.^43^	Japan	Retrospective	No	64	Open	7/16
2020	Napolitano *et al*.^39^	USA	Retrospective	No	524	Open	7/16
2022	Kempeneers *et al*.^38^	The Netherlands	Retrospective	No	70	Open	18/24
**Minimally invasive *versus* open LPJ**				(*n* = 102)			
2017	Kirks *et al*.^31^	USA	Retrospective	Yes	26	Robotic *versus* open	14/24
2022	Nag *et al*.^32^	India	Retrospective	Yes	76	Laparoscopic *versus* open	18/24

MIS, minimally invasive surgery; LPJ, lateral pancreaticojejunostomy; MINORS, Methodological Index for Non-Randomized Studies.

**Table 2:** Intraoperative outcomes of lateral pancreaticojejunostomy

Study	Operative time*		EBL†	
	MIS	Open	MIS	Open
Sielezneff *et al*.^41^	–	–	–	–
Sohn *et al*.^42^	–	312	–	600
Nealon and Matin^40^	–	–	–	–
Kalady *et al*.^28^	–	–	–	–
Boerma *et al*.^26^	–	S: 170 NS: 165	–	–
Nealon and Walser^33^	–	CJ: 147 CA: 95	–	–
Tantia *et al*.^37^	LPJ: 277	–	–	–
Palanivelu *et al*.^34^	179	–	–	–
Khaled and Ammori^29^	277	–	150	–
Sahoo and Kumar^35^	262	–	–	–
Sudo *et al*.^43^	227	227	–	298
Kirks *et al*.^31^	268	236	76	250
Kim and Hong^30^	201	–	42	–
Hamad *et al*.^27^	210	–	45	–
Bhandarwar *et al*.^25^	Staple: 171 Sutured: 213	Conversion: 185	–	–
Senthilnathan *et al*.^36^	221	–	184	–
Napolitano *et al*.^39^	–	241	–	–
Nag *et al*.^32^	300	210	100	120
Kempeneers *et al*.^38^	327	–	300	–

^*^Mean/median (min); †mean/median (ml); MIS, minimally invasive surgery; S, stent; NS, no stent; CJ, cystojejunostomy; CA, cyst aspiration.

**Table 3:** Postoperative outcomes of lateral pancreaticojejunostomy

Study	Morbidity rate†		Length of stay‡		Readmission rate (%)		Mortality rate (%)	
	MIS	Open	MIS	Open	MIS	Open	MIS	Open
Sielezneff *et al*.^41^	–	30	–	14	–	–	–	0
Sohn *et al*.^42^	–	31	–	12	–	–	–	2
Nealon and Matin^40^	–	4	–	–	–	–	–	0
Kalady *et al*.^28^	–	25	–	12	–	3	–	0
Boerma *et al*.^26^	–	S: 19 NS: 8	–	10	–	S: 26 NS: 33	–	0
Nealon and Walser^33^	–	CJ: 16 CA: 11	–	CJ 9 CA: 9	–	–	–	0
Tantia *et al*.^37^	12	–	5	–	–	–	0	–
Palanivelu *et al*.^34^	8	–	5	–	–	–	0	–
Khaled and Ammori^29^	20	–	5	–	20	–	0	–
Sahoo and Kumar^35^	0	–	8	–	–	–	0	–
Sudo *et al*.^43^	–	33	–	16	–	16	–	0
Kirks *et al*.^31^	57	68	5	7	14	16	0	0
Kim and Hong^30^	9	–	6	–	–	–	0	–
Hamad *et al*.^27^	25	–	7	–	38	–	0	–
Bhandarwar *et al*.^25^	29	–	6	Conversion: 8.5	–	–	0	–
Senthilnathan *et al*.^36^	POPF: 8 PPH: 3	–	6	–	–	–	0	–
Napolitano *et al*.^39^	–	35	–	10	–	10	–	1
Nag *et al*.^32^	8	10	7	6	–	–	0	0
Kempeneers *et al*.^38^	–	9	–	9	–	–	–	30-days: 1 90-day: 3

†Mean/median (ml); ‡mean/median (days); MIS, minimally invasive surgery; EBL, estimated blood loss; S, stent; NS, no stent; CJ, cystojejunostomy; CA, cyst aspiration; POPF, postoperative pancreatic fistula; PPH, postpancreatectomy haemorrhage.

All studies included patients with painful CP (*Table S2*). The most common aetiologies for CP were alcohol, tropical, and idiopathic. Postoperative outcome was reported by all studies whereas long-term outcomes such as pain relief, weight gain and new onset of exocrine or endocrine insufficiency were reported by 15, 5 and 7 studies respectively. Pain relief after surgery was evaluated using different methods (for example visual analogue scale, Izbicki pain score, complete or partial pain relief, etc.) and timing of follow-up (1, 2, 3, and 5 years) among studies. The studies by Kirks *et al.*^31^ and Kalady *et al.*^28^ were the only two reporting on costs.

### Short-term outcomes in MIS LPJ

Short-term outcomes in MIS LPJ are shown in *Tables 2*, *3*. Operative time per study ranged from 171 to 300 min and the estimated blood loss (EBL) ranged from 42 to 184 ml. Postoperative morbidity rate ranged from 0% to 57% (median and i.q.r. could not be calculated).

Length of hospital stay was from 5 to 7 days, readmission rate from 14% to 38% (median and i.q.r. could not be calculated) and mortality rate was 0% overall (median: 0, i.q.r.: 0).

### Short-term outcomes in open LPJ

Short-term outcomes in open LPJ are shown in *Tables 2*, *3*. Operative time varied from 95 to 327 min and EBL from 120 to 600 ml. Postoperative morbidity rate ranged between 4% and 68% (median: 25, i.q.r.: 23). Length of hospital stay ranged from 6 to 16 days. Readmission rate ranged from 3% to 33% (median: 16, i.q.r.: 16.5) and in-hospital mortality rate varied from 0% to 3% (median: 0, i.q.r.: 1).

### Long-term outcomes

Percentages of the longest follow-up period were used for the calculation of medians and i.q.r. of pain relief and endocrine and exocrine insufficiencies. The rate of pain relief ranged from 62% to 91% after open LPJ (median: 78.5, i.q.r.: 23) and from 71% to 100% (median: 82.5, i.q.r.: 12.5) after MIS LPJ respectively. New-onset endocrine insufficiency was reported in 21% to 22% (median and i.q.r. could not be calculated) in MIS LPJ and for 19% to 26% of patients after open LPJ. New-onset exocrine insufficiency was shown in 11% to 27% in MIS LPJ *versus* 8% to 26% after open LPJ (median and i.q.r. could not be calculated). Finally, the rate of weight gain ranged from 60% to 100% (median: 97, i.q.r. 23) after MIS LPJ (*Table S1*). Weight gain following open LPJ was not reported in the included studies.

### Costs

Two studies^28,31^ reported on hospital costs (*Table S1*). The first study reported a mean hospital cost for open LPJ of $13 530 whereas the latter compared median total cost between robotic and open LPJ (robotic LPJ $23 286 *versus* open LPJ $27 186)^31^. Additionally, a subanalysis revealed differences in costs for operative supplies (robotic LPJ $4063 *versus* open LPJ $1854) and postoperative medication (robotic LPJ $220 *versus* open LPJ $568)^31^.

### Methodological quality assessment

Individual MINORS scores in MIS LPJ and open LPJ were relatively low, ranging from 4 to 10 and 7 to 18 respectively. Studies comparing MIS LPJ and open LPJ had higher scores of 14 and 18. RoB2^24^ was not used as no randomized studies were included.

## Discussion

This systematic review on MIS *versus* open LPJ in patients with symptomatic CP suggests that, based on very low-quality evidence, MIS LPJ may hold some benefits in short-term morbidity rate and length of hospital stay. Long-term outcomes (for example pain relief, exocrine and endocrine insufficiency) appear to be similar between MIS and open LPJ. It must be emphasized that high-quality studies are needed, especially a phase 2 RCT which should also take postoperative pain control and costs into account.

As this is the first systematic review on this topic, these findings could not be compared to previous systematic reviews. Systematic reviews are available for MIS *versus* open pancreatoduodenectomy, confirming a shorter length of hospital stay with the MIS approach^44–46^. Compared to pancreatoduodenectomy, LPJ is associated with lower rates of major morbidity and mortality rates, which could be explained by the lower incidence of postoperative pancreatic fistula^16^ following LPJ relative to the hard pancreatic texture and atrophic parenchyma resulting from CP. Additionally, the main goal of surgery in CP is to increase QoL by reducing pain.

Because MIS is, in general, associated with less postoperative pain compared to open surgery, it is of paramount importance in the treatment of CP based on the challenges with pain therapy in those patients.

As a result of technological improvements and increasing experience with MIS, surgeons started performing MIS LPJ in the early 2000s. Some innovations have been reported since. In 2018, Bhandarwar *et al.*^25^ reported the feasibility of using staples for performing LPJ, thereby reducing operative time without worsening postoperative outcomes. Key to this technique is the presence of a wide main pancreatic duct to properly place the surgical stapler device. In 2017 the first series from the USA reported on robot-assisted MIS LPJ. Since then, two further studies reported on this approach (at least five patients). The robotic platform may be particularly useful given the extensive amount of suturing that is required during LPJ.

Only two studies directly compared outcome after MIS and open LPJ. These studies, from the USA^31^ and India^32^, reported largely similar postoperative outcomes after MIS and open LPJ. In a single-centre retrospective non-matched series of 26 patients, Kirks *et al*. reported lower EBL (75 ml *versus* 250 ml, *P* = 0.043) and length of stay (5 *versus* 7, *P* = 0.009) with robotic MIS LPJ compared to open, despite a 32 min increase in operative time (268 min *versus* 236 min, *P* = 0.412)^31^. Additionally, Kirks *et al*. reported reduced total cost with the robotic approach (Spearman’s rank correlation, robot, *r*: −0.775, *r*^2^: 0.600; open, *r*: −0.573, *r*^2^: 0.328). In a single-centre matched series of 76 patients, Nag *et al*. reported lower intraoperative blood loss (100 ml *versus* 120 ml, *P* = 0.009) with laparoscopic MIS LPJ as compared to open LPJ despite a 90 min increased operative time (300 min *versus* 210 min, *P* = 0.000). Notably, this study also reported a high conversion rate of 29%^32^. These findings should be assessed by further studies. The Miami guidelines for minimally invasive pancreatic surgery provide no guidance on MIS LPJ but advise a minimum volume of 20 MIS pancreatoduodenectomy procedures per centre^47^. Given the lower incidence of LPJ, these numbers are difficult to gather; hence, MIS LPJ might be reserved for high-volume centres, which have experience in both minimally invasive pancreatic surgery and minimally invasive pancreatic surgery specifically for CP.

A crucial point highlighted by the present systematic review was the lack of uniform reporting regarding the assessment of pain relief and the complete absence of reporting on postoperative (in hospital) pain control. The latter is of obvious importance as this would be an important mechanism for a benefit of MIS LPJ translating into faster time to functional recovery and shorter hospital stay. Regarding long-term pain relief, several definitions were used, making it impossible to perform a proper comparison. Standardized pain assessment with universal pain relief scores should be mandatory to properly understand the potential benefit of surgery in CP patients. In 2020, the Dutch Pancreatitis Study Group published the ESCAPE trial^8^, which reported a superiority of surgery as first intervention for CP compared to endoscopic treatment. Early surgery compared with an endoscopy-first approach resulted in lower pain scores over the course of 18 months. Three years later, Kempeneers *et al.*^48^ further reported results of the ESCAPE trial with early surgery being superior compared to the endoscopy-first approach from a cost-effectiveness point of view. Additionally, performing endoscopy prior to surgery decreases the benefit of surgery^49^. Based on these and other studies, the role of surgery as reference standard for chronic pancreatitis is now widely accepted. However, proper standardization of intraoperative management, postoperative management and follow-up is still lacking.

Several limitations should be considered when interpreting the results of this systematic review. There was considerable heterogeneity between the included studies in terms of outcome reporting, thereby making results difficult to interpret. Future studies should report complications in accordance with current guidelines (for example ISGPS definitions^16–21^) and define outcomes properly in the study methods. The study size varies remarkably between studies on MIS (median 12 patients) and open (median 64 patients) LPJ. This was driven by the inclusion criteria of minimum 5 MIS LPJ or 50 open LPJ, highlighting a significant bias related to the quality of data because the open group holds more data from high-volume centres and thus likely reporting better quality. Several included studies report follow-up times that are not adequate for patients with CP because pain relief is not always expected directly following surgery but is expected in the years after surgery, so future studies including a minimum of 12 months of follow-up are necessary. Although 177 patients after MIS LPJ were included in this review, only 15 patients underwent robot-assisted MIS LPJ. Cleary more data on this subgroup are needed. Literature comprised retrospective studies with a relatively low number of patients with CP and relatively low methodological quality resulting in a systematic review of low-quality evidence only. According to the IDEAL recommendations^50^, pragmatic phase 2 RCTs are now warranted to provide stronger evidence on the role of MIS LPJ in eligible patients with symptomatic CP. Most MIS LPJ studies (75%) were published after 2014 whereas the majority of open LPJ studies (67%) were published before 2004. This highlighted a strong temporal bias, as surgical tools and postoperative management have improved in the last decade.

In conclusion, this systematic review of low-quality evidence suggested that MIS LPJ can be performed safely in selected patients. The next step would be to perform a phase 2 RCT with standardized outcome measures focusing on potential short-term benefits for patients.

## Supplementary Material

zrae135_Supplementary_Data

## Data Availability

No new data were generated or analyzed in support of this research.
