# Supplementary material for: Minimally invasive versus open lateral pancreaticojejunostomy in patients with painful chronic pancreatitis: systematic review
Source: BJS Open. 2025 Jan 21;9(1):zrae135. doi: 10.1093/bjsopen/zrae135 (PMC11747668; doi:10.1093/bjsopen/zrae135)
Supplement: zrae135_Supplementary_Data [file zrae135_supplementary_data.zip › ST1.docx]

**Supplementary table 1. Long-term outcome of lateral pancreaticojejunostomy**

| **Study** | **Complete pain relief^*^** | | **New-onset endocrine insufficiency^*^** | | **New-onset exocrine insufficiency^*^** | | **Weight gain^*^** | | **Costs° ($)** | |
| --- | --- | --- | --- | --- | --- | --- | --- | --- | --- | --- |
|  | **MIS** | **Open** | **MIS** | **Open** | **MIS** | **Open** | **MIS** | **Open** | **MIS** | **Open** |
| Sielezneff et al. | - | 64 | - | - | - | - | - | - | - | - |
| Sohn et al. | - | - | - | - | - | - | - | - | - | - |
| Nealon et al. | - | 87 | - | - | - | - | - | - | - | - |
| Kalady et al. | - | - | - | - | - | - | - | - | - | 13.530 |
| Boerma et al. | - | - | - | - | - | - | - | - | - | - |
| Nealon et al. | - | 86 | - | - | - | - | - | - | - | - |
| Tantia et al. | 82 | - | - | - | - | - | 94 | - | - | - |
| Palanivelu et al. | 83 | - | - | - | - | - | 100 | - | - | - |
| Khaled et al. | 80 | - | - | - | - | - | 60 | - | - | - |
| Sahoo et al. | 83 | - | - | - | - | - | 100 | - | - | - |
| Sudo et al. | - | 91 | - | 19 | - | - | - | - | - | - |
| Kirks et al. | - | - | - | - | - | - | - | - | 23.286 | 27.186 |
| Kim et al. | 100 | - | - | - | - | - | - | - | - | - |
| Hamad et al. | - | - | - | - | - | - | - | - | - | - |
| Bhandarwar et al. | 88 | - | - | - | - | - | - | - | - | - |
| Senthilnathan et al. | 1 year: 90  3 year: 88  5 year: 88 | - | 1 year: 7  3 year: 14  5 year: 22 | - | 1 year: 7  3 year: 13  5 year:27 | - | - | - | - | - |
| Napolitano et al | - | - | - | - | - | - | - | - | - | - |
| Nag et al. | 71 | 66 | 21 | 26 | 11 | 8 | - | - | - | - |
| Kempeneers et al. | - | 62 | - | 24 | - | 26 | - | - | - | - |

** Percentage (%); ° Mean total cost;*
