# Supplementary material for: Minimally invasive versus open lateral pancreaticojejunostomy in patients with painful chronic pancreatitis: systematic review
Source: BJS Open. 2025 Jan 21;9(1):zrae135. doi: 10.1093/bjsopen/zrae135 (PMC11747668; doi:10.1093/bjsopen/zrae135)
Supplement: zrae135_Supplementary_Data [file zrae135_supplementary_data.zip › ST2.docx]

**Supplementary table 2. Studies populations**

| **Years** | **Authors** | **Study population** | |  |
| --- | --- | --- | --- | --- |
| **Minimally-invasive LPJ** | |  | |  |
| 2004 | Tantia et al. | Consecutive patients with an established diagnosis of chronic obstructive pancreatitis on radiological investigations | |  |
| 2006 | Palanivelu et al. | Patients with chronic calculous pancreatitis | |  |
| 2014 | Khaled et al. | Patients with established diagnosis of chronic pancreatitis by CT-scan and endoscopic retrograde cholangiopancreatography | |  |
| 2014 | Sahoo et al. | Patients with chronic pancreatitis | |  |
| 2016 | Kim et al. | Patients with chronic pancreatitis with pancreatic duct stones accompanied by signs of pancreatic duct obstruction | |  |
| 2017 | Hamad et al. | Patients with confirmed chronic pancreatitis as their primary diagnosis on final pathology | |  |
| 2018 | Bhandarwar et al. | Patients with a confirmed diagnosis of chronic pancreatitis with recurrent pain for 2 years or more | |  |
| 2019 | Senthilnathan et al. | Patients with chronic pancreatitis with unremitting abdominal pain | |  |
| **Open LPJ** | |  |  |  |
| 2000 | Sielezneff et al. | Patients with chronic alcoholic pancreatitis, defined as chronic pancreatitis associated with the consumption of more than 50 g of alcohol/day for at least five years, with no other aetiological factor for pancreatitis. | |  |
| 2000 | Sohn et al. | Patients with chronic pancreatitis | |  |
| 2001 | Nealon et al. | Patients with established diagnosis of chronic pancreatitis by CT-scan and endoscopic retrograde cholangiopancreatography | |  |
| 2001 | Kalady et al. | Patients with chronic pancreatitis | |  |
| 2002 | Boerma et al. | Patients with painful chronic pancreatitis in whom the presence of an inflammatory mass had been ruled out by imaging | |  |
| 2003 | Nealon et al. | Patients with chronic pancreatitis and main pancreatic duct diameter greater than 7 mm. | |  |
| 2014 | Sudo et al. | Patients with chronic pancreatitis patients with dilated main pancreatic duct. All patients were diagnosed with chronic pancreatitis by clinical history, ultrasonography, CT-scan, and endoscopic retrograde cholangiopancreatography | |  |
| 2020 | Napolitano et al. | Patients with chronic pancreatitis | |  |
| 2022 | Kempeneers et al. | Patients with an established diagnosis of chronic pancreatitis according the M-ANNHEIM diagnostic criteria | |  |
| **Minimally-invasive vs open LPJ** | | | |  |
| 2017 | Kirks et al. | Patients with chronic pancreatitis | |  |
| 2022 | Nag et al. | Patients with diagnosis of chronic pancreatitis based on CT-scan of the abdomen and histopathological examination | |  |
