# Supplementary material for: Minimally invasive versus open lateral pancreaticojejunostomy in patients with painful chronic pancreatitis: systematic review
Source: BJS Open. 2025 Jan 21;9(1):zrae135. doi: 10.1093/bjsopen/zrae135 (PMC11747668; doi:10.1093/bjsopen/zrae135)
Supplement: zrae135_Supplementary_Data [file zrae135_supplementary_data.zip › Supplementary_material.docx]

**Minimally-invasive versus open lateral pancreaticojejunostomy in patients with painful chronic pancreatitis: systematic review**

R.M. Montorsi^1, 2, 3*^, M.F.G. Francken^1, 2, 4*^, M.A. Boermeester^1,2^, O.R. Busch^1,2^, F. Daams^2,5^, T. Hackert^6^, R. Haen^7^, M.W. Hollmann^8^, H.C. van Santvoort^9, 10^, M.G. Besselink^1,2^ for the Dutch Pancreatitis Study Group
**Shared first authorship*

**Affiliations:**

^1^ Department of Surgery, Amsterdam UMC, location University of Amsterdam, Amsterdam, the Netherlands;

^2^ Amsterdam Gastroenterology Endocrinology Metabolism, Amsterdam, the Netherlands;

^3^ Department of General and Pancreatic Surgery, The Pancreas Institute, University of Verona Hospital Trust, Verona, Italy;

^4^ Department of Research and Development, St Antonius Hospital, Nieuwegein, the Netherlands;

^5^ Department of Surgery, Amsterdam UMC, location Vrije Universiteit, Amsterdam, the Netherlands;

^6^ Department of General, Visceral and Thoracic Surgery, University Medical Center Hamburg-Eppendorf, Hamburg, Germany;

^7^ Department of Surgery, Erasmus University Medical Center, Rotterdam, the Netherlands;

^8^ Department of Anesthesiology, Amsterdam UMC, location University of Amsterdam, Amsterdam, the Netherlands;

^9^ Department of Surgery, St. Antonius Hospital, Nieuwegein, the Netherlands;

^10^ Department of Surgery, University Medical Center Utrecht, Utrecht, the Netherlands;

**Corresponding author.** Roberto Maria Montorsi **ORCID ID** 0009-0003-6260-2269; **Twitter** R_Montorsi

**Supplementary Materials – Index**

| **Supplementary Figures and Tables** |  |
| --- | --- |
| Supplementary table 1. Long-term outcome of lateral pancreaticojejunostomy | *pag. 3* |
| Supplementary table 2. Studies populations | *pag. 4* |
| **References** | *pag. 5* |
|  |  |

**Supplementary Figures and Tables**

**Supplementary table 1. Long-term outcome of lateral pancreaticojejunostomy**

| **Study** | **Complete pain relief^*^** | | **New-onset endocrine insufficiency^*^** | | **New-onset exocrine insufficiency^*^** | | **Weight gain^*^** | | **Costs° ($)** | |
| --- | --- | --- | --- | --- | --- | --- | --- | --- | --- | --- |
|  | **MIS** | **Open** | **MIS** | **Open** | **MIS** | **Open** | **MIS** | **Open** | **MIS** | **Open** |
| Sielezneff et al. (1) | - | 64 | - | - | - | - | - | - | - | - |
| Sohn et al. (2) | - | - | - | - | - | - | - | - | - | - |
| Nealon et al. (3) | - | 87 | - | - | - | - | - | - | - | - |
| Kalady et al. (4) | - | - | - | - | - | - | - | - | - | 13.530 |
| Boerma et al. (5) | - | - | - | - | - | - | - | - | - | - |
| Nealon et al. (6) | - | 86 | - | - | - | - | - | - | - | - |
| Tantia et al. (7) | 82 | - | - | - | - | - | 94 | - | - | - |
| Palanivelu et al. (8) | 83 | - | - | - | - | - | 100 | - | - | - |
| Khaled et al. (9) | 80 | - | - | - | - | - | 60 | - | - | - |
| Sahoo et al. (10) | 83 | - | - | - | - | - | 100 | - | - | - |
| Sudo et al. (11) | - | 91 | - | 19 | - | - | - | - | - | - |
| Kirks et al. (12) | - | - | - | - | - | - | - | - | 23.286 | 27.186 |
| Kim et al. (13) | 100 | - | - | - | - | - | - | - | - | - |
| Hamad et al. (14) | - | - | - | - | - | - | - | - | - | - |
| Bhandarwar et al. (15) | 88 | - | - | - | - | - | - | - | - | - |
| Senthilnathan et al. (16) | 1 year: 90  3 year: 88  5 year: 88 | - | 1 year: 7  3 year: 14  5 year: 22 | - | 1 year: 7  3 year: 13  5 year:27 | - | - | - | - | - |
| Napolitano et al. (17) | - | - | - | - | - | - | - | - | - | - |
| Nag et al. (18) | 71 | 66 | 21 | 26 | 11 | 8 | - | - | - | - |
| Kempeneers et al. (19) | - | 62 | - | 24 | - | 26 | - | - | - | - |
|  |  |  |  |  |  |  |  |  |  |  |

** Percentage (%); ° Mean total cost;*

**Supplementary table 2. Studies populations**

| **Years** | **Authors** | | **Study population** | |  |
| --- | --- | --- | --- | --- | --- |
| **Minimally-invasive LPJ** | | |  | |  |
| 2004 | Tantia et al.(7) | | Consecutive patients with an established diagnosis of chronic obstructive pancreatitis on radiological investigations | |  |
| 2006 | Palanivelu et al.(8) | | Patients with chronic calculous pancreatitis | |  |
| 2014 | Khaled et al.(9) | | Patients with established diagnosis of chronic pancreatitis by CT-scan and endoscopic retrograde cholangiopancreatography | |  |
| 2014 | Sahoo et al.(10) | | Patients with chronic pancreatitis | |  |
| 2016 | Kim et al.(13) | | Patients with chronic pancreatitis with pancreatic duct stones accompanied by signs of pancreatic duct obstruction | |  |
| 2017 | Hamad et al.(14) | | Patients with confirmed chronic pancreatitis as their primary diagnosis on final pathology | |  |
| 2018 | Bhandarwar et al.(15) | | Patients with a confirmed diagnosis of chronic pancreatitis with recurrent pain for 2 years or more | |  |
| 2019 | Senthilnathan et al.(16) | | Patients with chronic pancreatitis with unremitting abdominal pain | |  |
| **Open LPJ** | | |  |  |  |
| 2000 | Sielezneff et al.(1) | | Patients with chronic alcoholic pancreatitis, defined as chronic pancreatitis associated with the consumption of more than 50 g of alcohol/day for at least five years, with no other aetiological factor for pancreatitis. | |  |
| 2000 | Sohn et al.(2) | | Patients with chronic pancreatitis | |  |
| 2001 | Nealon et al.(3) | | Patients with established diagnosis of chronic pancreatitis by CT-scan and endoscopic retrograde cholangiopancreatography | |  |
| 2001 | Kalady et al.(4) | | Patients with chronic pancreatitis | |  |
| 2002 | Boerma et al.(5) | | Patients with painful chronic pancreatitis in whom the presence of an inflammatory mass had been ruled out by imaging | |  |
| 2003 | Nealon et al.(6) | | Patients with chronic pancreatitis and main pancreatic duct diameter greater than 7 mm. | |  |
| 2014 | Sudo et al.(11) | | Patients with chronic pancreatitis patients with dilated main pancreatic duct. All patients were diagnosed with chronic pancreatitis by clinical history, ultrasonography, CT-scan, and endoscopic retrograde cholangiopancreatography | |  |
| 2020 | Napolitano et al.(17) | | Patients with chronic pancreatitis | |  |
| 2022 | Kempeneers et al.(19) | | Patients with an established diagnosis of chronic pancreatitis according the M-ANNHEIM diagnostic criteria | |  |
| **Minimally-invasive vs open LPJ** | | | | |  |
| 2017 | Kirks et al.(12) | Patients with chronic pancreatitis | | |  |
| 2022 | Nag et al.(18) | Patients with diagnosis of chronic pancreatitis based on CT-scan of the abdomen and histopathological examination | | |  |

*MIS:minimally-invasive surgery; LPJ: lateral pancreatico-jejunostomy; MINORS: [Methodological index for non-randomised studies](https://pubmed.ncbi.nlm.nih.gov/12956787/)*

**References**

1. Sielezneff I, Malouf A, Salle E, Brunet C, Thirion X, Sastre B. Long term results of lateral pancreaticojejunostomy for chronic alcoholic pancreatitis. Eur J Surg. 2000 Jan;166(1):58–64.

2. Sohn TA, Campbell KA, Pitt HA, Sauter PK, Coleman JA, Lillemo KD, et al. Quality of life and long-term survival after surgery for chronic pancreatitis. J Gastrointest Surg. 2000;4(4):355–64; discussion 364-365.

3. Nealon WH, Matin S. Analysis of surgical success in preventing recurrent acute exacerbations in chronic pancreatitis. Ann Surg. 2001 Jun;233(6):793–800.

4. Kalady MF, Broome AH, Meyers WC, Pappas TN. Immediate and long-term outcomes after lateral pancreaticojejunostomy for chronic pancreatitis. Am Surg. 2001 May;67(5):478–83.

5. Boerma D, van Gulik TM, Rauws EA, Obertop H, Gouma DJ. Outcome of pancreaticojejunostomy after previous endoscopic stenting in patients with chronic pancreatitis. Eur J Surg. 2002;168(4):223–8.

6. Nealon WH, Matin S. Analysis of surgical success in preventing recurrent acute exacerbations in chronic pancreatitis. Ann Surg. 2001 Jun;233(6):793–800.

7. Tantia O, Jindal MK, Khanna S, Sen B. Laparoscopic lateral pancreaticojejunostomy: our experience of 17 cases. Surg Endosc. 2004;18(7):1054–7.

8. Palanivelu C, Shetty R, Jani K, Rajan PS, Sendhilkumar K, Parthasarthi R, et al. Laparoscopic lateral pancreaticojejunostomy: a new remedy for an old ailment. Surg Endosc. 2006;20(3):458–61.

9. Khaled YS, Ammori BJ. Laparoscopic lateral pancreaticojejunostomy and laparoscopic Berne modification of Beger procedure for the treatment of chronic pancreatitis: the first UK experience. Surg Laparosc Endosc Percutan Tech. 2014 Oct;24(5):e178-182.

10. Sahoo M, Kumar A. Laparoscopic longitudinal pancreatico jejunostomy using cystoscope and ERCP basket for retrieval of left over pancreatic duct stones. Surgical Endoscopy and Other Interventional Techniques. 2013;27:S288.

11. Sudo T, Murakami Y, Uemura K, Hashimoto Y, Kondo N, Nakagawa N, et al. Short- and long-term results of lateral pancreaticojejunostomy for chronic pancreatitis: a retrospective Japanese single-center study. J Hepatobiliary Pancreat Sci. 2014 Jun;21(6):426–32.

12. Kirks RC, Lorimer PD, Fruscione M, Cochran A, Baker EH, Iannitti DA, et al. Robotic longitudinal pancreaticojejunostomy for chronic pancreatitis: Comparison of clinical outcomes and cost to the open approach. Int J Med Robot. 2017;13(3):e1832.

13. Kim EY, Hong TH. Laparoscopic Longitudinal Pancreaticojejunostomy Using Barbed Sutures: an Efficient and Secure Solution for Pancreatic Duct Obstructions in Patients with Chronic Pancreatitis. J Gastrointest Surg. 2016;20(4):861–6.

14. Hamad A, Zenati MS, Nguyen TK, Hogg ME, Zeh HJ, Zureikat AH. Safety and feasibility of the robotic platform in the management of surgical sequelae of chronic pancreatitis. Surg Endosc. 2018 Feb;32(2):1056–65.

15. Bhandarwar A, Arora E, Gajbhiye R, Gandhi S, Patel C, Wagh A, et al. Laparoscopic lateral pancreaticojejunostomy: an evolution to endostapled technique. Surg Endosc. 2019 Jun;33(6):1749–56.

16. Senthilnathan P, Subrahmaneswara Babu N, Vikram A, Sabnis SC, Srivatsan Gurumurthy S, An, et al. Laparoscopic longitudinal pancreatojejunostomy and modified Frey’s operation for chronic calcific pancreatitis. BJS Open. 2019;3(5):666–71.

17. Napolitano M, Brody F, Lee KB, Rosenfeld E, Chen S, Murillo-Berlioz AE, et al. 30-Day outcomes and predictors of complications after Puestow procedure. Am J Surg. 2020 Aug;220(2):372–5.

18. Nag H, Nekarakanti P, Arvinda P, Sharma A. Laparoscopic versus open surgical management of patients with chronic pancreatitis: Amatched case-control study. Journal of Minimal Access Surgery. 2022;18(2):191–6.

19. Kempeneers MA, van Hemert AKE, van der Hoek M, Issa Y, van Hooft JE, Nio CY, et al. Short- and long-term outcomes of selective use of Frey or extended lateral pancreaticojejunostomy in chronic pancreatitis. Br J Surg. 2022 Mar 15;109(4):363–71.
